# Neurological Pupillary Index (NPi) Measurement Using Pupillometry and Outcomes in Critically Ill Children

**DOI:** 10.7759/cureus.46480

**Published:** 2023-10-04

**Authors:** Jessie Jiang, Halil Sari, Rachelle Goldman, Erionne Huff, Ashley Hanna, Ravi Samraj, Hariprasad Gourabathini, Utpal Bhalala

**Affiliations:** 1 Medicine, Texas A&M College of Medicine, Round Rock, USA; 2 Statistics, Texas A&M College of Medicine, Round Rock, USA; 3 Pediatric Critical Care Medicine, Driscoll Children's Hospital, Corpus Christi, USA; 4 Pediatric Neurosurgery, Driscoll Children's Hospital, Corpus Christi, USA; 5 Pediatric Critical Care Medicine, Beacon Children's Hospital, South Bend, USA; 6 Pediatrics, Texas A&M College of Medicine, College Station, USA; 7 Anesthesiology and Critical Care, Driscoll Children's Hospital, Corpus Christi, USA

**Keywords:** outcome assessment, critical care, pupillary reflex, pediatrics, brain injuries

## Abstract

Aim/objective

Neurological Pupil Index (NPi), measured by automated pupillometry (AP), allows the objective assessment of pupillary light reflex (PLR). NPi ranges from 0 (non-reactive) to 5 (normal). In this study, we aimed to compare neurologic and functional outcomes in children admitted for neurologic injury with normal (≥3) versus abnormal (<3) NPi measured during their pediatric intensive care unit (PICU) stay.

Materials and methods

We conducted a retrospective chart review of children between one month and 18 years admitted to our PICU with a diagnosis of neurologic injury between January 2019 and June 2022. We collected demographic, clinical, pupillometer, and outcome data, including mortality, Pediatric Cerebral Performance Category (PCPC), Pediatric Overall Performance Category (POPC), and Functional Status Score (FSS) at admission, at discharge, and at the three to six-month follow-up. We defined abnormal pupil response as any NPi <3 at any point during the PICU stay. Using the student's t-test and chi-square test, we compared the short-term and long-term outcomes of children with abnormal NPi (<3) versus those with normal NPi (≥3).

Results

There were 49 children who met the inclusion criteria and who had pupillometry data available for analysis. The mean (SD) Glasgow Coma Scale (GCS) in the study cohort was 5.6 (4.3), and 61% had low (<3) NPi during ICU stay. Mortality was significantly higher among patients with an abnormal NPi as compared to those with normal NPi. Children with abnormal NPi exhibited significant worsening of neurologic and functional status (ΔPCPC, ΔPOPC, and ΔFSS) from admission to discharge (mean (SD): 3.55(1.5), 3.45(1.43), 16.75(7.85), p<0.001) as compared to those with normal NPi (mean (SD): 1.45(0.93), 1.73(0.90), 3.55(2.07), p>0.05). The significant difference in neurologic and functional status persisted at the three to six-month follow-up between the two groups - children with abnormal NPi (mean (SD): 2.0(1.41), 2.08(1.38), 6.92(6.83), p<0.01) and children with normal NPi (mean (SD): 0.82(1.01), 0.94(1.03), 1.53(1.70), p>0.05).

Conclusion

In our retrospective cohort study, children admitted to the PICU for a neuro injury and with abnormal NPi (< 3) have higher mortality, and worse short-term and long-term neurologic and functional outcomes as compared to those with normal NPi (≥ 3) measured during the PICU course. AP provides an objective assessment of PLR and has potential applications for neuro-prognostication. More research needs to be done to elucidate the prognostic value of NPi in pediatrics.

## Introduction

Neurocritically ill patients make up 16% of all Pediatric Intensive Care Unit (PICU) admissions. These patients have higher mortality and longer hospital stays, and survivors acquire more new morbidities as compared to general PICU admissions [1]. Clinical neuromonitoring, serial neurological exams in critically ill patients, is key to identifying deficits representing a new or evolving nervous system injury [2]. An important neuromonitoring exam is assessing the pupillary light reflex (PLR). The PLR describes the constriction and subsequent dilation of the pupil in response to light stimuli [3]. PLR is used for the prognosis of traumatic brain injuries, and loss of PLR is associated with neurological deterioration and poor neurologic outcomes. Traditionally, practitioners perform the assessment by shining a handheld light source in the patient’s eyes. They record the reflex status using terms like “brisk, reactive,” or “sluggish” and subjectively record pupil dimensions to be “3 mm” without using a measuring device. A study focused on manual bedside pupillometry found low inter-rater reliability between examiners and only 33.3% of the pupils scored as non-reactive by examiners were scored similarly by the automated pupillometer [4].

The introduction of an automated infrared pupillometer (AP) in the Intensive Care Unit (ICU) increases the accuracy and reliability of pupillary measurements [5]. AP uses infrared light to measure the baseline pupil size. Afterward, it flashes the eye with a 3-second burst of visible light and records the pupillary response using the device’s built-in camera. It quantifies components of PLR including percent change, the velocity of constriction from baseline following a light stimulus, latency, and dilation velocity after constriction response. Compiling all these factors and using the device’s patented algorithm, the pupillometer measures the PLR by assigning it a Neurological Pupil Index (NPi). NPi scores range from 0 to 5 with a normal response being ≥3 [6]. Any score <3 is considered an abnormal response and a score of zero is a non-reactive pupil and a score of 5 is a normal pupil [7]. It has been studied to have no adverse events when used on children of all ages, but its usage may be limited by patient cooperation in awake children [8].

AP research in adults supports a potential role in detecting significant intracranial events [9] and elevated intracranial pressure (ICP) [10]. Research also supports possible prognostication using NPi after cardiac arrest (CA) [11,12]. Even though there is ample literature on NPi in adults, research exploring NPi in children is insufficient. Published literature in children includes normative data on PLR reference values in healthy children [8], but investigations into AP use in critically ill children are limited to one study on its use in the detection of increased ICP [13]. Therefore, we conducted a retrospective study aimed at comparing outcomes in comatose children admitted for neurologic injury with normal (NPi ≥ 3) versus abnormal (NPi < 3) pupillometry measurements taken during their ICU stay. Our hypothesis is that lower NPi values in critically ill children correlate with worse outcomes.

## Materials and methods

Study design and patients

We performed a retrospective chart review on critically ill children admitted between January 2019 to July 2022 to the two PICUs at Driscoll Children’s Hospital and Beacon Children’s Hospital. Inclusion criteria for patients were if they were between 1 month to 18 years old, admitted to the PICU for moderate to severe neurologic injury defined as Glasgow Coma Scale (GCS) < 10 or rapid decline in GCS 24 hours from admission. Exclusion criteria included if there was no pupillometry data available. Neonates were excluded due to the PICU age limits. The study (Pupillometry in Pediatric Intensive Care Unit) was approved by the Institutional Review Board of Driscoll and Beacon Children’s Hospital with a waiver of informed consent in accordance with paragraph 26 of the Helsinki Declaration (approval date: 2/10/2022; approval number: 22.004).

Data and outcomes

We utilized the EPIC Slicer/Dicer application, a built-in search tool that allows you to filter patients by diagnosis and to screen patients with specific diagnostic terms such as cerebral edema, encephalopathy, hypoxic-ischemic encephalopathy, meningoencephalitis, brain tumor, stroke, cerebral/cerebellar hemorrhage, cerebrovascular accident, traumatic brain injury, or status epilepticus. Patients were grouped based on abnormal (NPi < 3) or normal (NPi ≥ 3) NPi measurements based on the lowest NPi recorded during their stay in the PICU. We collected various data, including demographics, clinical details, pupillometer readings, and outcomes from electronic medical records. Clinical information encompassed GCS, initial and lowest NPi values, and admission diagnosis.

The primary focus was to compare mortality between patients with abnormal and normal pupillometry findings during their PICU stay. Secondary outcomes encompassed measures like ventilator-free days, ICU and hospital length of stay (LOS), and changes (Δ) in neurologic and functional outcome scores from admission to discharge and follow-up. These outcome scores included pediatric cerebral performance category (PCPC), pediatric overall performance category (POPC), and functional status score (FSS), which measured change in neurologic, overall, and functional outcomes, respectively. PCPC and POPC are scored from 1 (normal) to 6 (brain dead). FSS assesses the patient in six categories (mental status, sensory, communication, motor function, feeding, and respiratory) and is scored from 6 (normal) to 30 (brain dead). The first author (JJ) underwent a training process supervised by the principal investigator (UB) to ensure accuracy in score measurement. Unfavorable outcomes were defined as PCPC or POPC ≥ 3 or FSS ≥ 12, based on a previous pediatric study [14].

Pupillometry measurements

**Figure 1:**
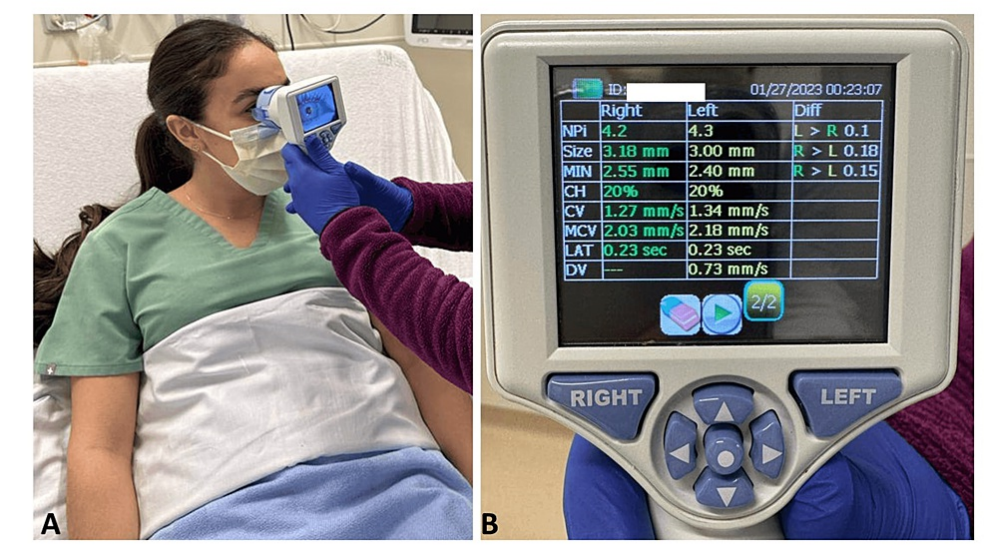
A. Nurse taking automated pupillometer reading; B. Automated pupillometer reading displaying neurologic pupillary index values NPi = Neurologic Pupillary Index, Size = Maximum Diameter, MIN = Minimum Diameter, CH = % Change, LAT = Latency of Constriction, MCV = Maxiumum Constriction Velocity, DV = Dilation Velocity

The NeurOptics Npi-200 Pupillometer (NeurOptics®, Irvine, CA, USA) is an automated handheld device that measures baseline pupillary size and PLR using infrared technology [14]. Once the pupillometer is positioned correctly, it elicits and records the PLR (Figure 1). Using its patented algorithm, the pupillometer assigns the PLR an objective neurological pupillary index value. NPi values range from 0 (non-reactive) to 5 (normal). An NPi value < 3 is considered abnormal and a value ≥ 3 is normal [15]. We averaged the NPi values measured between the right and left eye and used the lowest NPi measured during the PICU course for this study. In the study’s PICUs, the automated pupillometer was introduced in 2019 with initial and recurring training of the PICU nurses regarding its use. Neither of the PICUs had any unit-specific protocol related to monitoring the PLR using an automated pupillometer and/or management of children with a neurologic injury using pupillometer data. Pupillometry assessment using an automated pupillometer was solely at the discretion of the clinical team.

Data analysis

We expressed descriptive data for continuous and categorical variables as mean ± SD or number with percentage (%), respectively. We performed a student t-test for the quantitative data and a chi-square test of independence with Yate’s continuity correction for the qualitative data. P-values <0.05 were considered significant. We also calculated odds ratio (OR) statistics with Haldane-Anscombe correction. We used the Haldane-Anscombe correction because there was a zero cell in two of the contingency tables (e.g., POPC*NPi, mortality*NPi), which causes the OR to be undefined. This correction overcomes zero cells in 2x2 contingency tables by adding 0.5 to each cell in the contingency table prior to the calculation [16,17]. We did not use the correction when there was no zero cell in the contingency table (e.g., PCPC*NPi and FSS*NPi). Odds ratios were performed to determine the odds of an unfavorable outcome given an NPi < 3. ROC curve analysis was performed to determine an optimal cutoff value for unfavorable outcomes and mortality by selecting the cutoff with the highest sensitivity and specificity value. PCPC, POPC, and FSS scores were scored as either unfavorable outcomes (PCPC or POPC ≥ 3 or FSS ≥ 12) [18] or favorable outcomes (FO) for odds ratio and receiver operator curve (ROC) analysis. We performed all data analysis in R Program Version 4.2.2 (The R Foundation for Statistical Computing, Institute for Statistics and Mathematics, Vienna, Austria).

## Results

Study population

During the study period, 49 patients qualified for the study’s final analysis located from both children’s hospitals’ databases. Patient demographics are shown in Table 1. Thirty (61.2%) patients had an abnormal NPi reading during their PICU stay. The median (IQR) age was four years (1.1-13.5); the majority (n(%)) of patients were female (31 (63.2%)) and Hispanic (27 (84.3%)). The primary diagnosis (n(%)) leading to PICU admission was a traumatic brain injury (18 (36.7%)), followed by non-traumatic intracranial hemorrhage (13 (26.5%)), central nervous system infection (8 (16.3%)), hypoxic-ischemic encephalopathy following cardiac arrest (6 (12.2%)), status epilepticus (3 (6.1%)), and diabetic coma (1 (2%)). The median (IQR) admission GCS for the patients was three (3-6.25) indicating most patients were comatose upon PICU admission (Table 1).

**Table 1:** Patient demographic and clinical data *Patients with significant cerebral edema due to causes other than trauma, hemorrhage, infection, status epilepticus, and arrest were included in this category. One of the examples is cerebral edema due to diabetic ketoacidosis. ICP = intercranial pressure, GCS = Glasgow Coma Scale, PICU = pediatric intensive care unit, NPi = Neurologic Pupillary Index

	Abnormal NPI (< 3)	Normal NPI (>=3)	Total
	n= 30	n=19	N=49
Demographics			
Age in years (median (IQR))	3.5 (0.81-8.75)	4 (1.96-14.00)	4 (1.1-13.5)
Female sex, n (%)	16 (53.3)	8 (42.1)	31 (63.2)
Hispanic, n (%)	19 (63.3)	12 (63.1)	27 (84.3)
Primary Diagnosis n(%)			
Traumatic Brain Injury/Trauma	10 (33.3)	8 (42.1)	18 (36.7)
Intracranial Hemorrhage	9 (30.0)	4 (21.1)	13 (26.5)
Central Nervous System Infection	3 (10.0)	5 (26.9)	8 (16.3)
Status Epilepticus	3 (10.0)	0 (0.0)	3 (6.1)
Cardiac Arrest	4 (13.3)	2 (10.5)	6 (12.2)
Cerebral Edema*	1 (3.3)	0 (0.0)	1 (2.0)
Neurologic Status in PICU n(%)			
GCS Admission (mean ± SD)	5.10	6.37	5.6 (4.3)
Treatment Interventions n(%)			
Mannitol given, Y	12 (40.0)	8 (42.1)	20 (40.8)
Hypertonic saline given, Y	16 (53.3)	6 (31.6)	22 (44.9)
DHC performed, Y	8 (26.7)	6 (31.6)	14 (28.6)
ICP Monitoring, Y	11 (36.7)	8 (42.1)	19 (38.8)

Primary and secondary outcomes

There was a significant difference in mortality between the abnormal NPi cohort (18/30 (60%)) versus the normal NPi cohort (no mortality) (p<0.001) (Table 2). The chi-square value of 15.53 (p<0.001) was also significant with a significant correlation between the NPi and outcomes of mortality. The OR was 57.72 (95% CI (3.18-1046.33)) meaning that during the PICU stay, the odds of dying are 57.72 times as large with abnormal NPi (Table 3). We did not find any significant difference between the two groups for hospital length of stay (LOS), ICU LOS, or ventilator-free days, p>0.05 (Table 2).

**Table 2:** Primary and secondary outcome results *For follow-up, the n for each group changes due to losing patients due to death. n=12 for NPi<3 and n=17 for NPi >=3. LOS = length of stay; ICU = intensive care unit; IV = intravenous; ICP = intracranial pressure; Delta = change from admission to discharge or follow-up; PCPC = pediatric cerebral performance category; POPC = pediatric overall performance category; FSS = functional status score

Outcomes (mean ± SD)	Abnormal NPI (< 3) (n=30)	Normal NPI (>=3) (n=19)	p
Mortality n(%)	18 (60)	0 (0)	<0.001
Hospital LOS	18.5 (18.47)	23.89 (16.26)	0.15
ICU LOS	13.87 (15.12)	13.84 (9.98)	0.5
Ventilator-free days	10.30 (13.73)	15.63 (12.83)	0.09
IV mannitol n(%)	12 (40)	8 (42.11)	0.88
IV hypertonic saline n(%)	16 (53.33)	6 (31.58)	0.14
Decompressive hemicraniectomy n(%)	8 (26.67)	6 (31.58)	0.71
Invasive ICP monitoring n(%)	11 (36.67)	8 (42.10)	0.70
Delta PCPC discharge	3.67 (1.47)	1.47 (1.07)	<0.001
Delta POPC discharge	3.6 (1.43)	1.68 (1.11)	<0.001
Delta FSS discharge	17.57 (7.67)	3.95 (4.78)	<0.001
Delta PCPC follow-up*	2.0 (1.41)	0.82 (1.01)	0.01
Delta POPC follow-up*	2.08 (1.38)	0.94 (1.03)	0.01
Delta FSS follow-up*	6.92 (6.83)	1.53 (1.70)	0.002

**Table 3:** Chi-square test of independence and odds ratio statistics OR = odds ratio; PCPC = pediatric cerebral performance category; POPC = pediatric overall performance category; FSS = functional status scale *Haldane-Anscombe correction was applied

Outcome	Chi-square	df	p	OR
Mortality	15.53	1	<0.001	57.72*
PCPC	9.22	1	0.002	21.09
POPC	10.06	1	0.002	36.6*
FSS	13.24	1	<0.001	14.08

There was a significant worsening of short-term neurologic and functional status (ΔPCPC, ΔPOPC, and ΔFSS from admission to discharge) among children with abnormal NPi (mean (SD): 3.67(1.47), 3.6(1.43), 17.57(7.67), p<0.001) as compared to those with normal NPi (mean (SD): 1.47(1.07), 1.68(1.11), 3.95(4.78), p>0.05). The chi-square value of 9.22 (p<0.001) indicated that there was a significant association between NPi and PCPC. The OR was 21.09 (95% CI (2.35-188.72)) meaning that during the PICU stay, the odds of an unfavorable outcome are 21.09 times as large with abnormal NPi (Table 3). We also found that there was a significant association between NPi and POPC (chi-square=10.06, p=0.002) and between NPi and FSS (chi-square = 13.25, p<0.001). These findings indicated that both POPC and FSS were dependent on the NPi. The odds of an unfavorable outcome of POPC are 36.6 (95% CI (1.93-690.56)) times as large with abnormal NPi. The odds of an unfavorable outcome of FSS are 14.08 (95% CI (3.37-58.83)) times as large with abnormal NPi (Table 3).

The ROC curve for NPi showed excellent accuracy at predicting mortality, and unfavorable outcomes in PCPC, POPC, and FSS scores (AUC= 0.89, 0.88, 0.90, 0.86 95% CI (0.80-0.98, 0.77-0.99, 0.82-0.99, 0.76-0.96)) respectively (Table 4). The optimal NPi cutoff value for determining an unfavorable outcome or favorable outcome in PCPC, POPC, and FSS at discharge is 3.5, 3.50, and 3.1 (sensitivity 88.9% and specificity 80%, sensitivity 100% and specificity 78.6%; sensitivity 76.5% and specificity 81.2%) respectively (Table 4). The optimal cutoff NPi value for mortality was 0.50 (sensitivity 90.3% and specificity 77.8%) (Table 4).

**Table 4:** ROC curve to predict unfavorable outcomes ROC = receiver operating curve; AUC = area under the curve; NPi = Neurologic Pupillary Index; PCPC = pediatric cerebral performance category; POPC = pediatric overall performance category; FSS = functional status scale

Variable	AUC (95% CI)	NPi Cut-off Values	Sensitivity (%)	Specificity (%)
Mortality	0.89 (0.80-0.98)	0.53	90.3	77.8
PCPC	0.88 (0.77-0.99)	3.38	100	78.6
POPC	0.90 (0.82-0.99)	3.5	88.9	80
FSS	0.86 (0.76-0.96)	3.1	76.5	81.2

Out of the 31 patients who survived the hospital discharge, 12 from the abnormal NPi group and 17 from the normal NPi group followed up, so a total of 29 patients had assessment information available for follow-up analysis (up to six-month follow-up). There were persistently significantly worse long-term neurologic and functional outcomes (ΔPCPC, ΔPOPC, and ΔFSS from admission to six-month follow-up) among children with abnormal NPi (mean (SD): 2.0 (1.41), 2.08 (1.43), 6.92 (6.83), p<0.01) as compared to those with normal NPi (mean (SD): 0.82 (1.01), 0.94 (1.03), 1.53 (1.70), p>0.05) (Table 2).

## Discussion

This study is the first pediatric study assessing the association between abnormal neurologic pupillary index as measured by automated pupillometry and outcomes in critically ill children. Our study demonstrated that abnormal NPi was associated with higher mortality and worse neurologic and functional outcomes in children with neurologic disorders compared to those with normal NPi during the PICU course.

The pupillary light reflex is an essential component of neuromonitoring in neurocritically ill patients. With subjective measurements, there is limited inter-rater reliability [4] indicating the need to standardize pupillometry measurements. AP provides an objective pupillary assessment with good reliability and a low error rate [19]. Additionally, AP can be used to accurately assess PLR in pediatric critically ill patients regardless of opioid status [15]. One multicenter study, including 2140 adult patients, found that the mean admission NPi for neurocritically ill patients regardless of GCS status was > 4 [20]. Some attempts have been made to establish normative data in pediatric patients using AP, but there is a lack of sufficient data on NPi in critically ill children [8].

Our pediatric study corroborates many of the similar conclusions drawn from adult literature. Our study found that very low NPi (cutoff value of 0.53) at any point during the PICU course correlated with higher mortality rates. Similarly, in an adult study all patients with non-reactive pupils, NPi=0, after resuscitation from cardiac arrest passed away [11]. Luz et al. found that abnormal NPi taken early in the post-injury phase in adults with acute brain injury predicted higher mortality [21]. Also, like multiple adult studies [11,12,21-24], our study showed that abnormal NPi is correlated with poorer short-term and long-term neurologic and functional outcomes. Adults with abnormal NPi measured post six hours after resuscitation from cardiac arrest had the worst outcomes at discharge [11]. In adult TBI patients, lower NPi during their ICU course is correlated with worse neurologic outcomes at one month [21], three months [12], and six months [22]. Fewer adult studies looked at how abnormal NPi correlated with functional outcomes. Two adult studies used the Glasgow Outcome Scale (GOS) to measure functional outcomes and found that abnormal NPi measured during the ICU course correlated with worse functional outcomes at one month [23] and six months [24].

Prior studies have assessed a cut-off NPi value as a predictor of unfavorable neurologic outcomes. The following studies varied in how they defined unfavorable neurologic outcomes: Luz et al. and Romagnosi et al. used GOS ≤ 3, Park et al. used GOS≤ 2, and Riker et al. and Oddo et al. used CPC ≥ 3 [11,12,21-23]. We defined unfavorable neurologic outcomes as PCPC ≥3 [18]. Luz et al. and Park et al. each found optimal NPi cut-off to predict unfavorable neurologic outcome as < 3.4 (with sensitivity and specificity each 84-86%) [21,23]. Riker et al. found the optimal NPi cut-off to predict unfavorable neurologic outcomes is < 3.7 (sensitivity 60% and specificity 82%) [11]. Our finding of a cutoff NPi value of 3.5 to predict unfavorable neurologic outcomes at discharge (sensitivity 88.9% and specificity 80%) corroborates with the prior adult findings. However, our result differs from Oddo et al. and Romagnosi et al. who determined the optimal NPi to predict unfavorable neurologic outcomes is ≤ 2 (58% sensitivity vs. 100% specificity) and < 3 (34% sensitivity vs. 98% specificity), respectively [12,22]. This difference may be due to larger sample sizes in these studies and the timing of NPi assessment. More research needs to be done on critically ill children with a larger sample size and NPi assessed at specific time points in the PICU.

Previous pediatric studies have investigated PLR using conventional pupillometry and brain injuries. Several studies have shown that abnormal or absent PLR during a PICU course is significantly correlated with increased mortality [25-27] and unfavorable PCPC outcomes [27]. The presence of PLR before initiating therapeutic hypothermia among children after cardiac arrest was significantly associated with favorable neurologic outcomes at the six-month follow-up [28]. While these findings are useful, they are based on subjective measurements. AP is a novel and promising technology that objectively measures PLR allowing for more accurate and reliable results. Research surrounding automated pupillometry in critically ill pediatric patients is very limited. One prospective cohort study found that NPi measurements inversely correlated with increased ICP [13]. Additionally, there is one case study on an infant with intracranial hemorrhage that explores AP to diagnose anisocoria when manual examination failed due to disparity in assessment [29]. To our knowledge, our study is the first to report findings of NPi as measured by AP and neurologic and functional outcomes in neurocritically ill children. Based on our findings, AP is a novel technology that has great potential to be implemented for neuro-prognostication among critically ill children.

Pupillary exams, neuroimaging, electroencephalogram, and neuro-injury biomarkers such as neuron-specific enolase are being used for prognostication [30]. AP provides an objective assessment of pupillary response and therefore it has more clinical relevance. Early prognostication of poor neurologic recovery is useful for guiding patient treatment and counseling families. It can avoid futile treatments for patients thus alleviating the psychological toll on families. Our findings pave the path for future studies evaluating NPi and outcomes in critically ill children. Future research should aim at designing a prospective study with a large and multicenter sample size measuring NPi at admission, at 24 hours, and at 72 hours in critically ill patients to better determine the prognostic value of NPi in pediatrics.

Our study has many limitations to acknowledge. First, the small sample size and bicentric and retrospective nature of our study indicate a possible selection bias and the lack of availability of this technology in all PICUs limits the generalizability of our results. Second, we analyzed NPi as a categorical variable of abnormal versus normal, which limits the applicability of the results over the course of the patient's time in the PICU. Third, there was no unit-based protocol for using AP to monitor PLR and physicians were not blinded to the NPi data. Physicians could have possibly altered a patient’s treatment based on NPi measurements, which is a limitation of the retrospective nature of this study. However, we found no statistically significant difference between treatment interventions offered to children in the normal versus abnormal NPi groups (Table 2). Fourth, patients with abnormal NPi tend to have more severe neurological conditions, so it is difficult to elucidate a causal relationship between abnormal NPi and unfavorable outcomes. All that can be currently stated from our study is that there is a correlation between abnormal NPi and unfavorable outcomes.

## Conclusions

Our retrospective, bicentric study found that children admitted to the PICU for a neuro injury with abnormal NPi (< 3) have higher mortality and worse short-term and long-term outcomes compared to those with normal NPi (≥ 3) measured during the PICU course. These findings highlight the potential of AP as a valuable tool for prognostication and monitoring in critically ill children. To better determine the prognostic value of NPi in pediatrics, future research should aim at designing a prospective study with larger sample sizes and set time points for NPi measurement.
